# Kidney MRI Texture Analysis—A Universal Assessment of Kidney State and Function?

**DOI:** 10.3390/jcm15124770

**Published:** 2026-06-19

**Authors:** Marcin Majos, Artur Klepaczko, Katarzyna Szychowska, Weronika Banasik, Ludomir Stefanczyk, Ilona Kurnatowska

**Affiliations:** 1 Department of Normal and Clinical Anatomy, Medical University of Lodz, 90-419 Lodz, Poland; 2 Medical Electronics Division, Institute of Electronics, Lodz University of Technology, 90-924 Lodz, Poland; 3 Department of Internal Diseases and Transplant Nephrology, Medical University of Lodz, 90-419 Lodz, Poland; 4 I Department of Radiology and Diagnostic Imaging, Medical University of Lodz, 90-419 Lodz, Poland

**Keywords:** chronic kidney disease, DWI, magnetic resonance, multiparametric, T1-weighted images, T2-weighted images

## Abstract

**Introduction:** Currently, chronic kidney disease (CKD) is detected based on glomerular filtration rate (GFR), proteinuria levels or kidney biopsy. However, the development of MRI techniques and AI algorithms gives hope to the assessment of CKD activity and kidney function with profound MRI image analysis. **Methods:** MRI images from healthy volunteers with no history of CKD were compared with those from CKD patients who had undergone both kidney MRI and kidney biopsy; the latter group was also divided into two subgroups based on CKD histopathological activity. Patients from both groups were scanned using either a 1.5 T or 3 T MRI scanner following sequential allocation (nine healthy controls and 28 CKD patients and 11 healthy volunteers and 43 CKD patients respectively for each scanner). **Results:** The final algorithm based on T1-weighted, T2-weighted and DWI images was able to distinguish patients with sensitivity ranging 77.78–87.50%, specificity 86.67–94.12% and precision 77.78–87.50%. Features of T1-weighted images and of T2-weighted images were found to correlate strongly with GFR with coefficients ranging from −0.5922 to −0.7090 and from 0.6126 to 0.6380, respectively. **Conclusions:** MRI image texture analysis may be suitable for assessing CKD activity, irrespective of the type of MRI scanner used. Furthermore, MRI image texture features correlate with eGFR values.

## 1. Introduction

Due to its high prevalence and high cost, chronic kidney disease (CKD) represents one of the greatest burdens on global healthcare systems. Currently, it is estimated to affect approximately 10.6% of the European population, with this percentage predicted to rise as populations age and the effectiveness of CKD treatment improves [1,2].

Currently, the initial diagnosis and monitoring of disease progression rely heavily on laboratory tests, specifically on assessing glomerular filtration rate (GFR) and proteinuria levels [3]. However, while it is possible to detect CKD and identify its subsequent exacerbations using such simple parameters, neither test provides all the necessary information needed for effective treatment. More importantly, kidney biopsy is still needed to confirm the precise etiology in the initial diagnosis and to determine the level of activity in the event of an exacerbation [4].

While kidney biopsy can provide a wealth of information regarding the condition, it is an invasive technique whose common complications include renal hematomas, perirenal hematomas or arteriovenous fistula formation [5]. Hence, there remains a need for alternative diagnostic methods that can assess renal function in a way that is less burdensome for patients.

For this purpose, many attempts have been made to harness magnetic resonance imaging (MRI), the radiological technique with the highest tissue resolution [6]. While early diagnostic protocols based on MRI date back to the mid-1980s [7,8], it was not until the 2000s that research in this area intensified [9]. In one of the pioneering trials in the field, Gillis et al. [10] analyzed T1-weighted images and arterial spin labeling in determining eGFR. In their study, designed on MRI images of 24 healthy volunteers and 17 CKD patients they found correlation between eGFR with T1-weighted images signal values in cortex (r = −0.75, *p* < 0.001) and between eGFR with estimated renal perfusion counted on the basis of arterial spin labeling (r = 0.73, *p* < 0.01). The team lead by Vijinder [11] undertook a study determining utility of ADC maps in evaluating CKD. On large group of 120 patients they proved that signals intensities of ADC maps can be used to stratify patients to CKD severity stages as their values differ statistically.

More recent studies have examined the potential of machine learning trained on T1-weighted kidney MRI images [12] and artificial intelligence trained on T2-weighted kidney MRI images from patients with CKD [13]. The aim of the present article is to determine the value of analyzing the textures of T1-weighted images, T2-weighted images and ADC maps in assessing CKD activity, and to confirm whether MRI features correlate with eGFR values in patients. Most notably, the study used an innovative approach by training artificial intelligence algorithms on images obtained from one MRI scanner and verifying them using images obtained from another.

## 2. Material and Methods

### 2.1. Subjects

The study used MRI images from two groups of participants. The first group consisted of healthy volunteers with no history of kidney disease (Group 1). The remainder consisted of Nephrology Clinic patients who underwent an MRI of the kidneys 24 h before or 24 h after a kidney biopsy due to an exacerbation of CKD. In our patient group, all kidney biopsies were performed on the right kidney. This second group was divided into two subgroups based on the results of histopathological examination:

Group 2—patients in the active phase of CKD who, on histopathological examination, exhibit signs of active disease, i.e., features of inflammatory infiltration—had a presence of lymphocytes and macrophages accompanied with present mesangial proliferation;

Group 3—patients in the inactive phase of CKD who exhibit irreversible, chronic histopathological changes such as matrix fibrosis and glomerular sclerosis, without visible histopathological features of inflammatory infiltration—had a presence of less than 2 lymphocytes or macrophages in sight with sclerosis of more than 80% of glomeruli.

Before the patients underwent kidney biopsy, GFR was assessed based on creatinine level thus: eGFRcr = 142 × min(Scr/κ, 1)α × max(Scr/κ, 1) − 1.200 × 0.9938 Age × 1.012 [if female]. (Scr—serum creatinine level).

Detailed demographic characteristics and group sizes are set out in Table 1 and Table 2.

CKD etiologies of analyzed patients are presented in Table 3.

In Group 1, i.e., healthy volunteers, images of both kidneys were included in the analysis. In groups 2 and 3, i.e., patients with CKD images of both kidneys were included if the MRI scan was performed prior to biopsy. If the MRI scan was performed after biopsy, only images of the non-biopsied kidney were included.

### 2.2. MRI Protocol

MRI scans were performed on two scanners using dedicated renal protocols. The first was a 3 T Magnetom Vida unit (Siemens Healthcare GmbH, Erlangen, Germany): sequence of dixon-dependent T1-weighted images (TR = 4 ms, TE1 = 1.26 ms, TE2 = 2.4 ms, TA = 14.78 ms) and T2-weighted HASTE images with fat saturation (TR = 1350 ms, TE = 80 ms, TA = 0.51 ms) and ADC map (TR = 1350 ms, TE = 80 ms, TA = 0.51 ms). The second was a 1.5 T Magnetom AvantoFit unit (Siemens Healthcare GmbH, Erlangen, Germany): sequence of dixon-dependent T1-weighted images (TR = 240 ms, TE1 = 2.37 ms, TE2 = 4.76 ms, TA = 20 ms) and T2-weighted HASTE images with fat saturation (TR = 1800 ms, TE = 95 ms, TA = 17.5 × 2) and ADC map (TR = 2000 ms, TE = 55 ms, TA = 1.38 ms).

The patients were allocated to a specific scanner on a first-come, first-served basis: the first patients underwent scans on the 3 T scanner, and once the planned number of scans had been reached, subsequent patients were scanned using the 1.5 T scanner. The images obtained using the 3 T scanner were used to train the algorithm, which was validated using the images from the 1.5 T scanner. The patients were allocated to MRI scanners sequentially.

The study was approved by the Bioethics Committee affiliated with the Medical University of Lodz, approval no. RNN/206/20/KE, dated 8 September 2020.

### 2.3. CKD Classification

The aim of the study was to design a kidney-state classification algorithm based on MRI images from three groups of patients: (1) patients without CKD, (2) patients with active lesions, (3) patients with chronic lesions. The images were obtained from two MRI scanners: 3 T and 1.5 T. In both cases, the imaging protocols included the same three methods: T1-weighted Dixon-Vibe imaging, T2-weighted imaging, and DWI. Due to the higher quality of the images obtained using the 3 T device, this data was solely used for classifier model training, with 5-fold cross-validation. The images from the 1.5 T scanner were used solely as a test set.

The classifier training procedure was as follows. In the feature extraction step, 221 texture features were calculated for each imaging sequence. The extracted features included texture models such as the image histogram, an autoregressive model, intensity gradient statistics, a greyscale run-length matrix, and a greyscale co-occurrence matrix. Next, the feature values were standardized to mean = 0 and variance = 1. In the standardized feature space, significant feature selection was performed: first separately within each imaging sequence, and then in the full multiparametric space. Feature selection was carried out using the sequential floating-forward selection (SFFS) algorithm and an SVM (support vector machine) classifier with a radial basis function. The SVM model parameters were chosen experimentally (C = 1, gamma = 1/*n*, where *n* is the number of features). Finally, the texture features selected as significant for the 3 T images were also computed and standardized (according to the mean and variance parameters from the 3 T data) for the 1.5 T images.

These features defined were thus used to train the final classifiers. For the 3 T images, 5-fold cross-validation was used, i.e., training was repeated five times, each time setting aside 20% of the data for testing. In contrast, for the 1.5 T images, the entire 3 T dataset was used for training.

### 2.4. GFR Predicting Model

To develop a predictive model of glomerular filtration rate based on texture parameters, a statistical modeling pipeline incorporating nested cross-validation (CV) was implemented to ensure unbiased model evaluation and prevent information leakage. The dataset consisted of 45 patients and 657 extracted radiomic features extracted from T1-weighted opposed-phase (T1OP), T1-weighted in-phase (T1IP), and T2-weighted kidney MR images. The wide GFR range enabled assessment of predictive performance across a broad spectrum of renal function.

The modeling process followed a two-level cross-validation strategy. In the outer loop (5 folds), the dataset was partitioned into training and test subsets to evaluate generalization performance. Within each outer training set, an inner loop (3-fold CV) was used exclusively for feature selection. Specifically, a two-stage feature selection procedure was applied: (1) correlation screening, where the top 50 features were preselected based on Pearson correlation with the target variable, and (2) forward stepwise selection, in which features were iteratively added based on improvement in cross-validated R^2^ score, up to a predefined maximum of 10 features.

A multivariate linear regression model was then fitted using the selected features within each fold, and performance was evaluated on the held-out outer test set. This nested design ensured that both feature selection and model fitting were conducted strictly within the training data of each fold, thereby avoiding optimistic bias.

To assess model assumptions, residuals from out-of-fold predictions were aggregated and evaluated for normality (Shapiro–Wilk test), homoscedasticity (Breusch–Pagan test), and independence (Durbin–Watson statistic). Additionally, feature selection stability was analyzed by tracking the frequency of each feature across outer folds. All statistical analyses were implemented in Python 3.14.6 programming language using standard statistical libraries, and significance was assessed at a two-sided α level of 0.05. Following nested CV, a final model was constructed using the subset of features most consistently selected across folds, and its performance was further evaluated using standard cross-validation.

To illustrate the impact of multidimensional modeling, univariate analysis was also performed within the same nested cross-validation framework. For each outer fold, the top features based on Pearson and Spearman correlation coefficients with the target variable were identified using training data only. Features were ranked based on the magnitude of their correlation coefficients, and statistical significance was assessed using corresponding *p*-values. To account for multiple comparisons across 657 features, Bonferroni correction was applied. Feature selection frequency across folds was then computed to assess the stability of correlation-based feature importance. Eventually, to evaluate the predictive contribution of individual radiomic parameters, single-variable linear regression models were constructed for the five most stable features identified through correlation-based ranking. For each single-feature model, the coefficient of determination (R2), adjusted R2, root mean squared error (RMSE), and cross-validated R2 were calculated. Cross-validation was used to estimate generalizable performance and to assess model stability between data splits.

### 2.5. Study Outcomes

Hence, the primary outcome of this study was the classification of a kidney-state based on CKD radiomic features. In addition, it was renal function, quantified by predicted glomerular filtration rate (GFR), which was treated as a continuous variable and modeled using linear regression.

## 3. Results

### 3.1. Texture-Based CKD Activity Classification

Finally, a relevant set of texture features was identified for analysis. All the selected features are listed in Table 4. These are grouped according to the algorithm used, viz. based on T1-weighted, T2-weighted or DWI images, or an algorithm based on all of the above sequences. A detailed explanation of the texture feature names can be found in the qMazDa manual [14].

Detailed metrics evaluating the validation of SVM models are given in Table 5, accompanied by the corresponding error tables (Table 6, Table 7, Table 8 and Table 9).

### 3.2. Texture-Based GFR Prediction

The study cohort exhibited a wide distribution of kidney function, with GFR values ranging from severe impairment to near-normal levels (Table 10). This heterogeneity provided a suitable basis for developing and evaluating texture-based prediction models.

Univariate correlation analysis demonstrated strong associations between several texture features and GFR. Many top-ranked features showed absolute Pearson and Spearman correlation coefficients exceeding 0.65, with highly significant *p*-values even after Bonferroni correction (Table 11 and Table 12). Most strongly correlated features originated from T1OP images and were negatively associated with GFR; in contrast the leading T2-weighted features correlated positively with GFR. Pearson and Spearman rankings were highly consistent, suggesting predominantly linear relationships between texture features and renal function.

**Figure 1:**
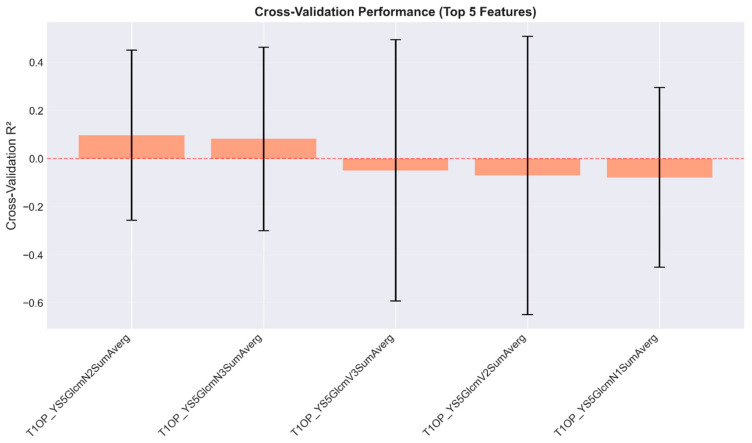
The orange bars represent mean R^2^ values across cross-validation folds. The black lines are the error bars extending between ±1 standard deviations of cross-validation scores. Error bars extend from negative to positive values, meaning the model performed acceptably in some CV folds, but performed worse than the predicted mean of the others.

**Figure 2:**
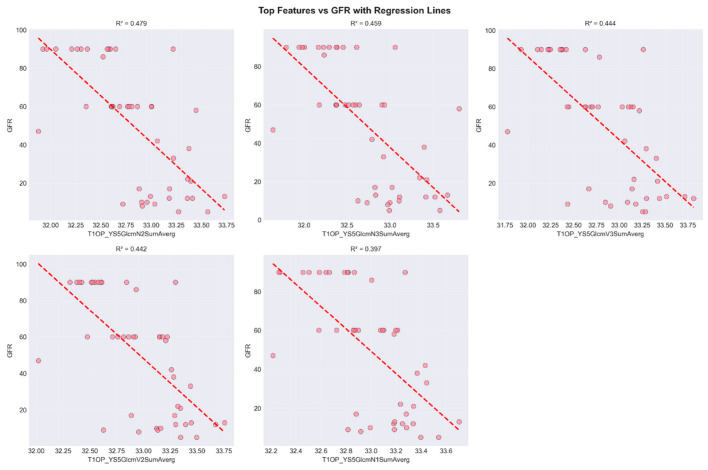
Actual vs. predicted GFR for 5 top features from correlation analysis.

By limiting the frequency of feature selections across CV folds to four, the intersection of rankings obtained using Pearson and Spearman correlations leads to a subset of five most stable GFR predictors (see Table 13). Single-feature linear regression models explained a moderate proportion of GFR variance in the training data, with maximum R2 values approaching 0.48 (Table 12). However, cross-validated R2 values were substantially lower and in some cases negative; these indicate limited model stability and suggest that individual texture features alone were insufficient for reliable prediction. This behavior is illustrated in Figure 1, where considerable variability was observed across cross-validation folds. The agreement between measured and predicted GFR for the strongest individual features is shown in Figure 2. The results reveal considerable dispersion around the identity line.

Multivariate modeling and simultaneous incorporation of radiomic features from various MRI sequences substantially improved regression accuracy. Overall, the nested cross-validation procedure demonstrated moderate predictive performance, with outer-fold R^2^ scores of 0.179, 0.655, 0.487, 0.119, and 0.649, yielding a mean R^2^ of 0.418 (±0.228). However, two folds achieved R^2^ values exceeding 0.64, indicating strong predictive capability in subsets of the data.

**Figure 3:**
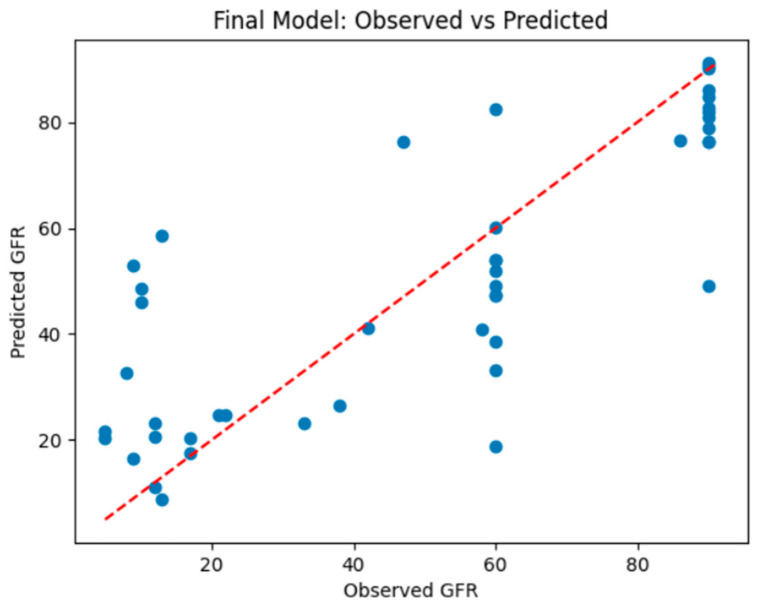
Actual vs. predicted GFR for the final multi-dimensional linear regression model.

Two-stage feature selection analysis revealed a consistent pattern, with five radiomic features repeatedly selected across folds. The most stable features included T1OP_YS5GlcmN2SumAverg and T2_YS5GlcmN3ClustPrm (selected in 4 out of 5 folds), as well as T2_YS5GlcmV2DifVarnc, T1OP_YS5GlcmH2SumAverg, and T1OP_YS5GlcmZ3AngScMom (selected in 3 out of 5 folds). These features were subsequently used to construct the final regression mode, whose performance, evaluated in a standard 5-fold cross-validation, reached R2 = 0.531 (± 0.168). This final model shows relatively good agreement between predicted and measured GFR values (Figure 3).

Based on the most frequently selected features, the final linear regression model was defined as:


$$\begin{array}{c} \mathrm{GFR}=1388.28-38.67\;(\mathrm{T1OP\_YS5GlcmN2SumAverg})-0.043\;(\mathrm{T2\_YS5GlcmN3ClustPrm})\\ -1.496\;(\mathrm{T2\_YS5GlcmV2DifVarnc})-1.920\;(\mathrm{T1OP\_YS5GlcmH2SumAverg}) \end{array}$$

All coefficients were negative, indicating that increased values of these texture features are associated with lower predicted GFR.

Residual diagnostics confirmed that model assumptions were adequately satisfied. Residuals followed a normal distribution (Shapiro–Wilk *p* = 0.956), exhibited no significant heteroscedasticity (Breusch–Pagan *p* = 0.237), and showed no strong autocorrelation (Durbin–Watson = 1.42).

**Figure 4:**
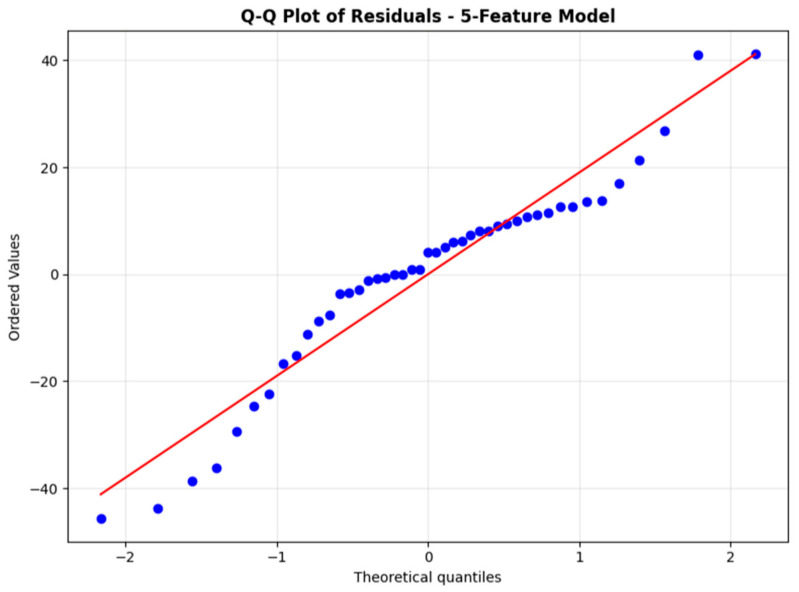
The Q–Q plot of the regression residuals. The ordered residuals align closely with the theoretical quantiles along the reference line, demonstrating that the residual distribution is approximately normal. Minor deviations are visible at the extreme tails, suggesting slight heavy-tailed behavior; however, these departures are small and not indicative of systematic non-normality. Neither skewness nor substantial outlier influence is present in the residuals, as confirmed by the absence of pronounced curvature or S-shaped patterns. Overall, the plot provides strong evidence that the residuals conform adequately to normality. As such, parametric inference based on the designed model appears valid.

Model diagnostics confirmed that the assumptions of linear regression were satisfied. Residuals were normally distributed, as supported by the Q–Q plot in Figure 4, exhibited constant variance, and showed no evidence of autocorrelation. Overall, these results demonstrate that a multi-parametric texture-based linear model can capture meaningful variation in renal function and outperform single-feature approaches. The findings support the use of combined MRI texture information for image-based assessment of chronic kidney disease.

## 4. Discussion

The present study took an innovative approach to creating a diagnostic algorithm: two sets of images were taken using two different scanners with different acquisition protocols; one set was used to create an algorithm, which was then validated using the second set. Hence, the proposed method should not be dependent on the examination protocol or the magnetic field strength generated by the MRI scanner. Despite this, it should only be regarded as preliminary, and requires verification in multicenter studies based on larger patient cohorts.

Nevertheless, Hara et al. [15] trained a precise algorithm to classify patients into groups according to the stage of CKD progression based on T1-weighted, T2-weighted and ADC maps. Similarly, Zhang et al. [16] trained an algorithm that also classified patients according to CKD stage using T1-weighted, T2-weighted and DWI images. These studies differ slightly in methodology from the present work, as they analyze patients in the inactive phase of CKD and are based on images acquired using only a single MRI scanner. Even so, they still confirm that texture features may serve as sensitive biomarkers for the progression of CKD.

Previous studies have examined the utility of texture analysis in assessing CKD. While they did not determine disease activity, they analyzed the effectiveness of texture-based algorithms in classifying patients according to disease severity.

For example, Shimizu et al. [17] trained models based on DWI in CKD of various etiology. In their trial, they divided study group of 68 patients into three categories based on eGFR levels (severe renal dysfunction—corresponding to CKD stages G4-G5, moderate renal dysfunction—corresponding to CKD stages G3a-G3b, mild renal dysfunction—corresponding to CKD stages G1-G2). Basing this solely on DWI, they succeeded to create algorithm utilizing 93 texture features which were characterized by an area under the curve AUC = 0.851 ± 0.010. Another team, Zhang et al. [18] used T2-weighted and ADC images in CKD associated with diabetes. Their study group consisted of 55 patients diagnosed with stage III diabetic nephropathy and 33 healthy volunteers. In their study they created three algorithms trained on fat saturated T2-weighted images, ADC and fat saturated T2-weighted images + ADC. Their models reached impressive AUC values of the test set; they were 0.91, 0.89 and 0.93 respectively with specificity and accuracy values of the united model were 0.90 and 0.89, respectively. Lastly, Yu et al. [19] reached an interesting outcome using T1-weighted, T2-weighted and DWI images in patients with CKD due to diabetes. The aim of the study was to determine if it is possible to distinguish healthy volunteers from patients suffering from stage III diabetic nephropathy. The analysis was made with MRI images derived from examinations of 44 healthy volunteers and 40 diabetic patients and the final model was characterized by predictive efficacy of 0.9486–1.0, specificity of 0.97 and precision of 0.93, which outperformed even the excellent model of Zhang et al. Their trial proved the potential of image texture analysis specifically of models trained on more than one sequence.

While these reports focus on GFR values rather than CKD stage, they still provide important evidence of the value of MRI texture analysis in the diagnosis and monitoring of CKD.

Our findings also indicate that numerous MRI image texture features correlate with GFR results; more precisely, orderliness within 5-pixel ROIs decreases as renal function deteriorates. These results are in line with previous studies of renal image textures, despite the fact that they did not examine linear correlation with GFR values as the studies focused on classifying patients into groups based on GFR range. We demonstrated that radiomic features derived from T1- and T2-weighted imaging provide moderate predictive value for GFR, explaining approximately 42% of its variance. This performance is notable given the small sample size and high dimensionality of the feature space, and it supports the hypothesis that imaging-derived texture descriptors capture biologically relevant information related to renal function.

Previous research on renal MRI compared the relationships between detected signal values and renal function. Buchanan et al. [20] noted negative correlations between T1-weighted and DWI signal values in both the renal cortex and medulla; interestingly, the reported correlation coefficients are very similar to those obtained in the present study. In addition, Lee et al. [21] note a correlation between corticomedullary differentiation indicated by T1-weighted images and single kidney glomerular filtration rate (SKGFR); the cohort consisted of a small group of patients who underwent renal MRI and 99mTc-diethylene triamine pentaacetic acid (DTPA) renography on the same day. In a large study of 506 patients by Lunzer et al. [22] GFR was found to correlate with T1-weighted signal values with a correlation coefficient of −0.701, i.e., very similar to our present results. Other reports have correlated T1-weighted signal values with CKD severity according to KIDGO [23]. While these studies are more common than typical linear correlation analyses, their findings only indirectly support our present observations [24,25,26,27].

Surprisingly, the textural characteristics of T2-weighted images were found to correlate with GFR, which contradicts previous research [25,28,29]. Our results may be confounded by the presence of renal edema and inflammation in patients in Group 2, who had higher GFR values than those in Group 3. Therefore, this observation requires further investigation and verification in a differently constructed study group.

Some reports indicate a correlation between DWI signal values and GFR [30,31], and suggest that DWI images may have prognostic value in the postoperative assessment of declining renal function [32]. Our data suggest that DWI images may correlate less strongly with renal function parameters than T1-weighted or T2-weighted images; however, our study group may have been too small to confirm these relationships. Also, our present findings were based on the textural characteristics of DWI images while the previous studies used exact DWI signal values.

Our study has limitations arising from the method of patient recruitment. Firstly, the study group is small. As the patients referred to our center often required urgent treatment, it was not always possible to perform an MRI scan within the strict timeframe. For the same reason, not all patients could be prepared for the MRI scan; for example, their hydration was not monitored. Also, another possible limitation is sequential allocation of patients to MRI scanners. It is possible that this method of qualification has not influenced the outcomes of the manuscript but for scientific clarity patients should have been randomly selected for MRI scanners.

Furthermore, GFR prediction model performance varied across folds, indicating sensitivity to sample composition, likely due to the limited dataset size. Also, while linear regression assumptions were met, the moderate R^2^ suggests that additional nonlinear relationships may remain unexplored. Thus, future work should focus on external validation, and exploration of nonlinear modeling approaches. Expanding the dataset would also improve model stability and enable more reliable feature selection.

Eventually, bilateral kidneys from the same patient were treated as separate observations in CKD classification as part of the analysis. This was done to increase sample size but might have introduced dependency and potentially biased the results. Given the larger sample, future studies should also incorporate patient-level aggregation or statistical methods accounting for within-subject correlation.

Lastly, our study is based on the assumption that histopathological changes are symmetrical in both kidneys. In systematic diseases, such as IgA nephropathies or lupus nephritis, this assumption should be correct; however, evaluating one kidney on the basis of the histopathological outcome of the other can lead to methodological bias.

Therefore, it is necessary to state that our study is preliminary and final conclusions of texture usefulness need further validation on larger groups. Also, our method demands more profound biological examination as we can state that image analysis has the possibility to differentiate patients between our groups; however, the precise histopathological background still has to be determined.

## 5. Conclusions

The analysis of MRI image textures may be of value for assessing disease activity in CKD; however, they still need histopathological verification during kidney biopsy. Our results suggest that the method may also be independent of the type of MRI scanner used; however, this conclusion needs to be verified in larger multicenter studies. They also indicate that MRI image texture features correlate with eGFR values.

## Figures and Tables

**Table 1:** Basic demographic characteristics of patients who underwent MRI examinations on 3 T MRI scanner.

Group	N	Sex		Mean Age in Years (Age Range)	Mean eGFR in mL/min/1.73 m^2^ (eGFR Range)
		Female	Male		
1	11	7	4	43.22 (29–64)	Above 60
2	29	16	13	50.46 (24–82)	42.90 (9–64)
3	14	6	8	56.16 (23–72)	26.60 (9–86)

**Table 2:** Basic demographic characteristics of patients who underwent MRI examinations on 1.5 T MRI scanner.

Group	N	Sex		Mean Age in Years (Age Range)	Mean eGFR in mL/min/1.73 m^2^ (eGFR Range)
		Female	Male		
1	9	5	4	43.44 (32–65)	Above 60
2	16	6	10	51.29 (21–71)	44.35 (8–84)
3	12	6	6	55.00 (41–78)	29.10 (7–89)

**Table 3:** Precise CKD etiologies of all patients divided by the scanner used for examination.

CKD Etiology	Group Scanned on 1.5 T MRI Scanner		Group Scanned on 3 T MRI Scanner	
	Group 2	Group 3	Group 2	Group 3
Focal segmental glomerular sclerosis	4	2	9	4
Vasculitis	2	1	4	0
Lupus nephritis	1	1	5	1
Tubulointerstitial nephritis	4	3	2	2
IgA nephropathy	3	1	4	2
Membranous nephropathy	2	1	5	0
Diabetes related nephropathy	0	1	0	1
End-stage kidney	0	2	0	4

**Table 4:** Selected feature in each imaging method.

T1-Weighted	T2-Weighted	DWI	All Modalities
T1IP_GlcmN1SumAverg T1OP_HistPerc99 T1OP_GlcmH2SumAverg T1OP_GlcmH3SumAverg T1OP_GlcmV2SumAverg T1OP_GlcmZ1ClustPrm T1OP_GlcmZ2SumAverg T1OP_GlcmN2SumAverg T1OP_YS5GlcmN3ClustPrm	GrlmVRLNonUni GlcmH1ClustPrm GlcmH3ClustPrm GlcmV2ClustShd GlcmV3ClustPrm GlcmZ1SumAverg GlcmZ1ClustShd GlcmZ2ClustPrm GlcmN2ClustPrm GlcmN3ClustPrm	HistMaxm01 HistDomn01 GrlmVGLevNonUn GrlmZLngREmph GrlmZShrtREmp GrlmZFraction GrlmZMRLNonUni GrlmNGLevNonUn GlcmH2ClustPrm GlcmZ1InvDfMom	ADC_GrlmZShrtREmp ADC_GrlmZMRLNonUni ADC_GlcmH3ClustPrm ADC_GlcmV2DifVarnc T1OP_HistMaxm01 T1OP_GlcmV2SumAverg T1OP_GlcmZ1ClustPrm T1OP_GlcmN3ClustPrm T2_HistPerc10 T2_GlcmV1SumAverg T2_GlcmV2SumVarnc T2_GlcmV3SumVarnc

**Table 5:** Metrics of all proposed algorithms.

		True Positive Ratio	Specificity	False Positive Ratio	Precision	F1
Algorithm based on T1-weighted image	Group 1	0.7222	0.9286	0.0714	0.8667	0.7879
	Group 2	0.8125	0.8667	0.1333	0.7647	0.7879
	Group 3	0.8333	0.8824	0.1176	0.7143	0.7692
Algorithm based on T2-weighted image feature metrics	Group 1	0.6667	0.9286	0.0714	0.8571	0.7500
	Group 2	0.8125	0.8000	0.2000	0.6842	0.7429
	Group 3	0.7500	0.8824	0.1176	0.6923	0.7200
Algorithm based on DWI image feature	Group 1	0.5556	0.8571	0.1429	0.7143	0.6250
	Group 2	0.7500	0.7333	0.2667	0.6000	0.6667
	Group 3	0.6667	0.8824	0.1176	0.6667	0.6667
Algorithm based on all sequences	Group 1	0.7778	0.9286	0.0714	0.8750	0.8235
	Group 2	0.8750	0.8667	0.1333	0.7778	0.8235
	Group 3	0.8333	0.9412	0.0588	0.8333	0.8333

**Table 6:** Confusion matrix of algorithm based on T1-weighted features.

	Predicted Group 1	Predicted Group 2	Predicted Group 3
True Group 1	13	3	2
True Group 2	1	13	2
True Group 3	1	1	10

**Table 7:** Confusion matrix of algorithm based on T2-weighted features.

	Predicted Group 1	Predicted Group 2	Predicted Group 3
True Group 1	12	4	2
True Group 2	1	13	2
True Group 3	1	2	9

**Table 8:** Confusion matrix of algorithm based on DWI features.

	Predicted Group 1	Predicted Group 2	Predicted Group 3
True Group 1	10	6	2
True Group 2	2	12	2
True Group 3	2	2	8

**Table 9:** Confusion matrix of algorithm based on combined T1-weighted, T2-weighted and DWI features.

	Predicted Group 1	Predicted Group 2	Predicted Group 3
True Group 1	14	3	1
True Group 2	1	14	1
True Group 3	1	1	10

**Table 10:** Presentation of study group heterogenicity according to texture features and GFR values.

Parameter	Value	Remarks
Sample size	45	Adequate for 5-feature model (9:1 ratio)
Features	657	Texture data—T1w (opposed- and in-phase), T2w
GFR range	5–90	Wide range of kidney function
GFR mean ± SD	49.8 ± 32.1	Heterogeneous population

**Table 11:** Top 10 texture features identified by Pearson correlation with GFR, with selection frequency across five cross-validation folds and cross-validated correlation coefficients (mean ± SD).

Feature	Correlation	*p*-Value	No. Occurrences Across 5 CV Folds
T1OP_YS5GlcmN2SumAverg	−0.6924 (0.0557)	*p* < 0.0001	5/5
T1OP_YS5GlcmN3SumAverg	−0.6795 (0.0449)	*p* < 0.0001	5/5
T1OP_YS5GlcmV3SumAverg	−0.6683 (0.0650)	*p* < 0.0001	5/5
T1OP_YS5GlcmV2SumAverg	−0.6649 (0.0722)	*p* < 0.0001	4/5
T1OP_YS5GlcmN1SumAverg	−0.6289 (0.0714)	*p* < 0.0001	4/5
T2_YS5GlcmH3SumAverg	0.6123 (0.0444)	*p* < 0.0001	3/5
T1OP_YS5GlcmZ2SumAver	−0.5998 (0.0581)	*p* < 0.0001	3/5
T1OP_YS5GlcmH2SumAverg	−0.5965 (0.0669)	*p* < 0.0001	2/5
T1OP_YS5GlcmH3SumAverg	−0.5908 (0.0656)	*p* < 0.0001	2/5
T2_YS5GlcmH2SumAverg	0.5786 (0.0489)	*p* < 0.0001	3/5

**Table 12:** Top 10 texture features identified by Spearman correlation with GFR, with selection frequency across five cross-validation folds and cross-validated correlation coefficients (mean ± SD).

Feature	Correlation	*p*-Value	No. Occurrences Across 5 CV Folds
T1OP_YS5GlcmN3SumAverg	−0.7091 (0.0547)	*p* < 0.0001	5/5
T1OP_YS5GlcmN2SumAverg	−0.7050 (0.0635)	*p* < 0.0001	4/5
T1OP_YS5GlcmV3SumAverg	−0.6759 (0.0670)	*p* < 0.0001	4/5
T1OP_YS5GlcmV2SumAverg	−0.6698 (0.0666)	*p* < 0.0001	4/5
T1OP_YS5GlcmN1SumAverg	−0.6423 (0.0906)	*p* < 0.0001	3/5
T2_YS5GlcmZ3SumAverg	0.6310 (0.0342)	*p* < 0.0001	3/5
T2_YS5GlcmH3SumAverg	0.6310 (0.0590)	*p* < 0.0001	2/5
T2_YS5GlcmH1SumAverg	0.6209 (0.0449)	*p* < 0.0001	3/5
T2_YS5GlcmZ1SumAverg	0.6277 (0.0392)	*p* < 0.0001	2/5
T1OP_YS5GlcmH2SumAverg	−0.6175 (0.0752)	*p* < 0.0001	3/5

**Table 13:** Linear regression single-feature models.

Feature	R^2^	Adj R^2^	RMSE	CV R^2^
T1OP_YS5GlcmN2SumAverg	0.479	0.466	22.94	0.097
T1OP_YS5GlcmN3SumAverg	0.459	0.447	23.36	0.082
T1OP_YS5GlcmV3SumAverg	0.444	0.432	23.68	−0.049
T1OP_YS5GlcmV2SumAverg	0.442	0.429	23.74	−0.071
T1OP_YS5GlcmN1SumAverg	0.397	0.383	24.67	−0.078

## Data Availability

Source code is available upon request to interested researchers from corresponding author.
